# Characterization of Polysaccharides with Antioxidant and Hepatoprotective Activities from the Edible Mushroom *Oudemansiella radicata*

**DOI:** 10.3390/molecules22020234

**Published:** 2017-02-04

**Authors:** Qin Liu, Mengjuan Zhu, Xueran Geng, Hexiang Wang, Tzi Bun Ng

**Affiliations:** 1 Institute of Plant Nutrition, Agricultural Resources and Environmental Science, Henan Academy of Agricultural Sciences, Zhengzhou 450002, China; liuqin_bio@hotmail.com; 2 State Key Laboratory for Agrobiotechnology, Department of Microbiology, China Agricultural University, Beijing 100193, China; 3 Department of Fungal Resource, College of Plant Protection, Shandong Agricultural University, Tai’an 271018, China; mengfan7777@163.com; 4 College of Food Science and Engineering, Shanxi Agricultural University, Taigu 030801, China; gengxueran2007@163.com; 5 School of Biomedical Sciences, Faculty of Medicine, The Chinese University of Hong Kong, Shatin, Hong Kong 999077, China

**Keywords:** antioxidant, hepatoprotective, *Oudemansiella radicata*, polysaccharides

## Abstract

The preliminary structure, in vitro antioxidant and in vivo hepatoprotective activities of water-soluble polysaccharides (ORWP) and alkali-soluble polysaccharides (ORAP), prepared from the mushroom *Oudemansiella radicata*, were investigated. Both ORWP and ORAP were heteropolysaccharides with mannose, glucose and galactose being the main monosaccharide components. Regarding the antioxidant activities, ORWP and ORAP showed effective 1,1-diphenyl-2-picrylhydrazyl (DPPH) radical scavenging activity, hydrogen peroxide scavenging activity and lipid peroxidation inhibitory effects, as well as moderate reducing power and Fe^2+^ chelating activity. For the hepatoprotective activity, administration of ORWP and ORAP prevented the increase in serum alanine aminotransferase and aspartate aminotransferase activities in a carbon tetrachloride-induced acute liver damage model, suppressed hepatic malondialdehyde formation and stimulated the activities of hepatic superoxide dismutase and glutathione peroxidase. Thus, we speculate that ORWP and ORAP may protect the liver from CCl_4_-induced hepatic damage via antioxidant mechanisms.

## 1. Introduction

The liver is a vital organ with detoxifying functions [1]. Many compounds, including clinically active drugs, can be metabolically activated into free radicals leading to oxidative stress [2]. CCl_4_ is used experimentally to induce acute liver injury. It is metabolized by the cytochrome P450 enzyme system into highly reactive trichloromethyl free radicals (•CCl_3_) and then to trichloromethylperoxy radicals (CCl_3_OO•), which attack biological molecules leading to lipid peroxidation and depletion of antioxidant enzymes [3]. Some drugs targeting the liver have potential adverse effects, especially when administered chronically [4]. For this reason, more attention has been paid to natural antioxidants believed to be harmless and free from adverse effects as an alternative therapy for liver diseases [5].

Recently, researchers have focused on mushrooms which produce a wide range of interesting bioactive compounds including lectins, ribonucleases, laccases, proteases, and polysaccharides [6,7]. These compounds exhibit antioxidative, antiproliferative, antitumor, immunomodulatory, and HIV-1 reverse transcriptase inhibiting activities with therapeutic potential [8]. Polysaccharides with various activities are produced by plants, bacteria, fungi, and algae [2,9,10,11]. Plant polysaccharides possessed antioxidant and anticancer activities [11]. Mushroom polysaccharides and polysaccharopeptides exhibit a diversity of activities. For instance, polysaccharide fractions of *Phellinus linteus* augmented cytokine production by macrophages and enhanced the cytotoxic action of natural killer cells [10], while ReishiMax, a mixture of polysaccharides and triterpenes from the medicinal mushroom *Ganoderma lucidum*, hampered adipocyte differentiation and stimulated glucose uptake by 3T3-L1 adipocytes [12]. Additionally, polysaccharopeptide from *Coriolus versicolor* displayed anticancer activity [13], and polysaccharide from *Pleurotus eryngii* exhibited antihyperlipidemic activities [7]. Furthermore, polysaccharides from some mushrooms exerted protective effects against acute hepatotoxicity in rats [14,15]. These activities endow mushrooms with a tremendous potential as a source of health-promoting compounds [15].

*Oudemansiella radicata*, an edible wild mushroom with high economic value, is distributed in broad-leafed forests. However, publications on this mushroom are confined to its antifungal antibiotic (the metabolite oudenone), and the effects of lead and cadmium on the mushroom [16,17]. As far as we know, there are no reports on the hepatoprotective effect of the polysaccharides from this mushroom against CCl_4_-induced hepatotoxicity in rats. In view of the scarce information and due to our observation that *O. radicata* polysaccharides displayed antioxidative activity, we hypothesized a possible protective effect of *O. radicata* on CCl_4_-induced liver injury.

## 2. Results

### 2.1. Isolation and Characterization of Polysaccharides

The yields of ORWP and ORAP were 7.59% and 5.01% of the dried fruiting bodies, respectively. The total carbohydrate contents of ORWP and ORAP were 95.3% and 97.4%, respectively. ORWP and ORAP consisted of ribose:rhamnose:arabinose:xylose:mannose:glucose:galactose with molar ratios of 0.15:0.32:1.02:2.45:15.74:65.85:14.47 and 0.16:0.51:1.02:3.31:18.41:72.35:4.24, respectively, but the monosaccharide composition of ORWP was identical to that of ORAP. The molecular weights of ORWP and ORAP, as determined by HPGPC, were 1.73 × 10^5^ and 1.15 × 10^4^ Da, respectively (Table 1). Negative results in the Bradford’s test and the lack of absorption at 280/260 nm indicated the possible absence of protein and nucleic acids.

**Figure 1:**
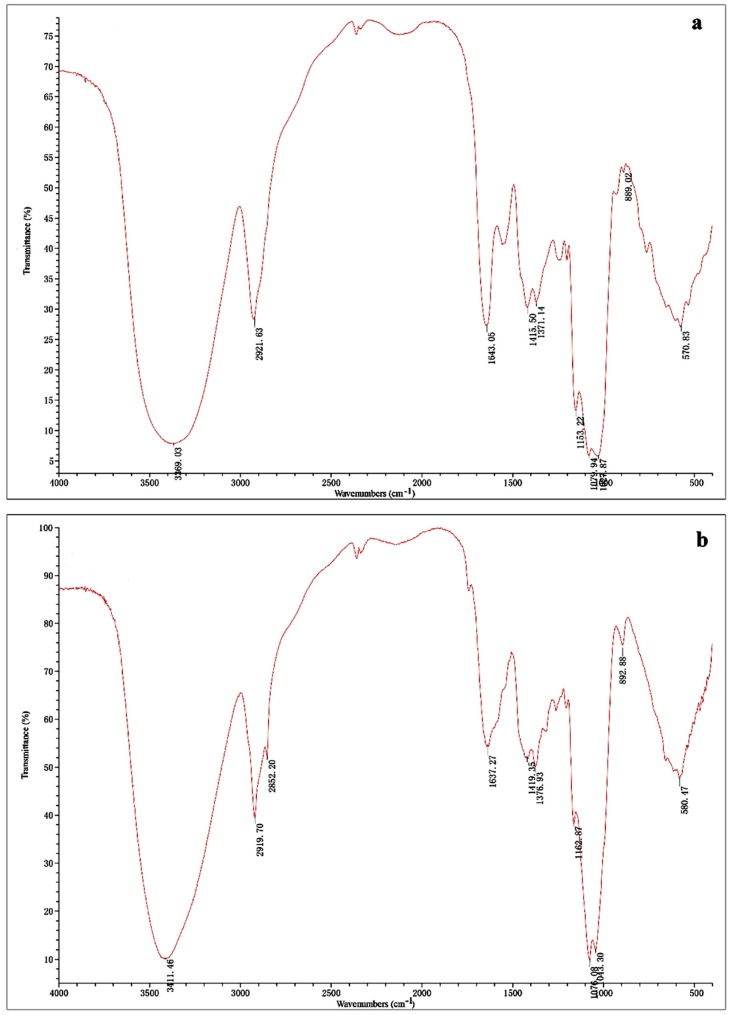
FT-IR spectrum of (**a**) ORWP and (**b**) ORAP.

As shown in Figure 1, the IR spectra of ORWP (Figure 1a) and ORAP (Figure 1b) were basically indistinguishable, with only some differences in the intensity of bands recorded from 4000 to 500 cm^−1^. Both ORWP and ORAP presented IR spectrum bands at 3600–3200 cm^−1^ (O-H stretching vibration) and 2900–2800 cm^−1^ (C-H stretching vibration), which are characteristic absorptions of polysaccharides [18]. The band at 1640 cm^−1^ was due to the presence of bound water and the band in the region of 1420 cm^−1^ was assigned to C-H bending vibrations [19]. The three bands at 1170–1025 cm^−1^ indicated the pyranose configurations of polysaccharides, and the characteristic absorptions at 890 confirmed the existence of β-glycosidic bonds [20].

### 2.2. In Vitro Antioxidant Activity

Five parameters were monitored and the results are as follows.

#### 2.2.1. DPPH Radical Scavenging Activity

**Figure 2:**
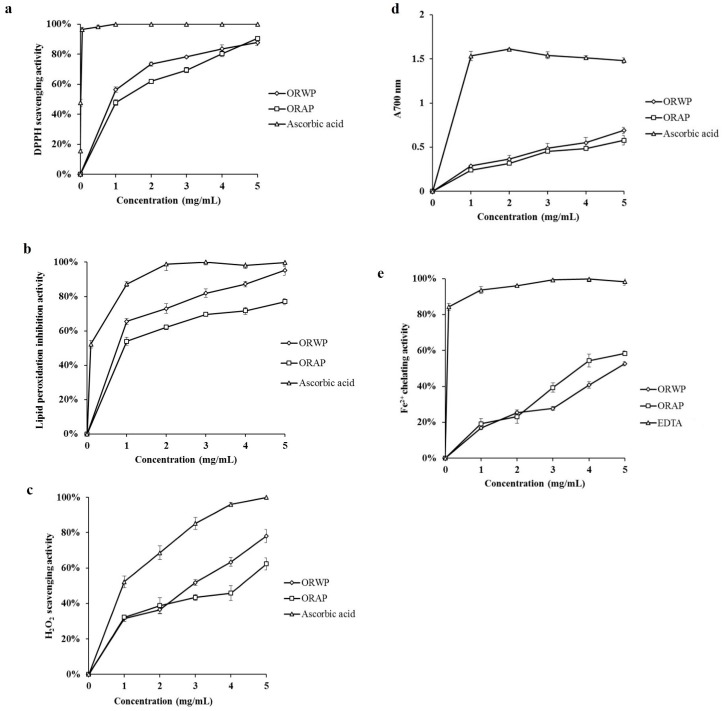
Antioxidant activity of ORWP and ORAP. (**a**) scavenging of DPPH radicals; (**b**) inhibitory effect on lipid peroxidation; (**c**) hydrogen peroxide scavenging activity; (**d**) reducing power; and (**e**) Fe^2+^ ion chelating activity. The values are representative of three separate experiments.

ORWP and ORAP exhibited potent DPPH scavenging activity, with IC_50_ = 0.78 ± 0.015 mg/mL and 1.25 ± 0.047 mg/mL, respectively. ORWP and ORAP at 5 mg/mL concentration scavenged 87.74% and 90.47% of DPPH radicals, respectively (Figure 2a).

#### 2.2.2. Inhibitory Effect on Lipid Peroxidation

Using the conjugated diene method, ORWP, ORAP and ascorbic acid at 1 mg/mL concentration produced respectively 65.56%, 53.92% and 87.24% inhibition of lipid peroxidation, thus showing that ORWP had a stronger inhibitory potential than ORAP, especially at 5 mg/mL. The IC_50_ values for ORWP, ORAP and ascorbic acid were 0.77 ± 0.013, 0.82 ± 0.012 and 0.12 ± 0.04 mg/mL, respectively (Figure 2b).

#### 2.2.3. Hydrogen Peroxide Scavenging Activity

At 5 mg/mL, ORWP, ORAP and ascorbic acid respectively scavenged 78.07%, 62.37% and 100% hydrogen peroxide. The IC_50_ values for ORWP, ORAP and ascorbic acid were 2.38 ± 0.12, 3.70 ± 0.64 and 1.28 ± 0.04 mg/mL, respectively (Figure 2c).

#### 2.2.4. Reducing Power

The reducing power of ORWP and ORAP increased dose-dependently (Figure 2d), but even at 5 mg/mL, ORWP and ORAP showed weaker reducing power than ascorbic acid.

#### 2.2.5. Ferrous Ion Chelating Activity

As shown in Figure 2e, the ferrous ion chelating activity of the extracts increased dose-dependently. When the concentration of samples ranged from 1 to 5 mg/mL, the Fe^2+^ ion chelating activity ranged from 16.90% to 52.67% chelation for ORWP, and from 19.15% to 58.34% chelation for ORAP. However, while EDTA showed an excellent Fe^2+^ chelating activity with 84.35% chelation at 0.1 mg/mL, ORWP (IC_50_ = 5.78 ± 0.30 mg/mL) and ORAP (IC_50_ = 3.93 ± 0.15 mg/mL) exhibited moderate chelating activity.

### 2.3. Animal Experiments

#### 2.3.1. Effects of Polysaccharides on Body Weight, Liver Weight and HI in Mice

The liver weight and HI of the mice increased significantly after CCl_4_ treatment compared with the normal control (peanut oil only). ORWP and ORAP at 100 and 200 mg/kg and bifendate at 200 mg/kg significantly prevented increases in liver weight and HI relative to the CCl_4_ group (Table 2). The body weights of the groups did not differ.

#### 2.3.2. Effects of the Polysaccharides on AST, ALT, MDA, GSH-Px and SOD Activities

**Figure 3:**
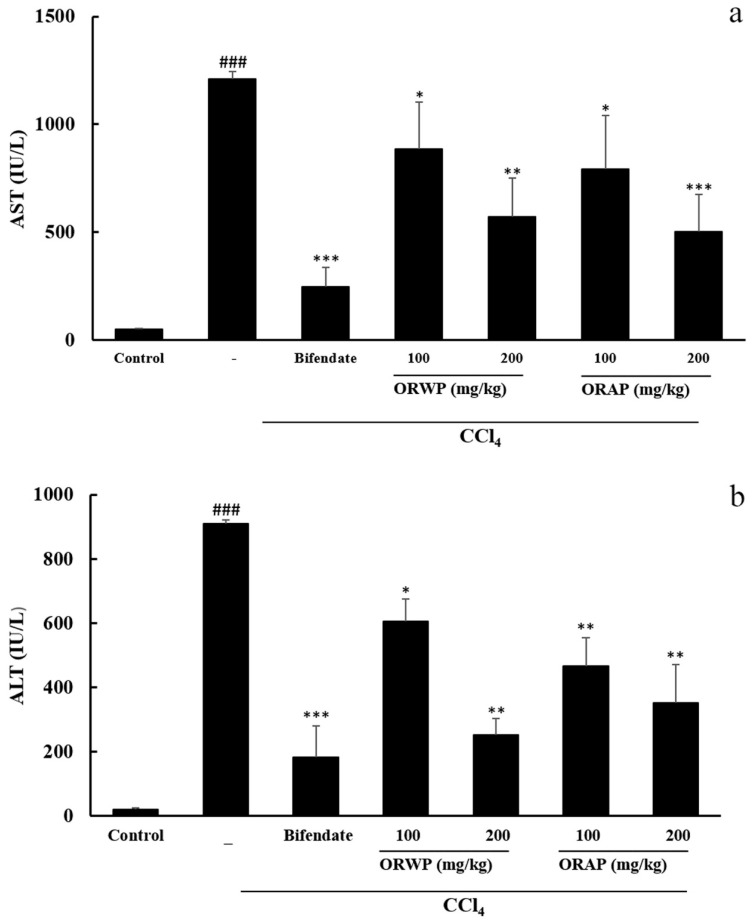
Effects of ORWP and ORAP on the activity of serum AST (**a**) and ALT (**b**) in CCl_4_-treated mice. The values represent means ± SD of eight mice/group. ^###^ *p* < 0.001 versus normal control, *** *p* < 0.001, ** *p* < 0.01 and * *p* < 0.05 versus CCl_4_-intoxicated group.

As displayed in Figure 3a,b, CCl_4_ treatment significantly increased the activities of AST and ALT in serum. When compared with the normal control group, mice treated with CCl_4_ alone showed acute liver damage as evidenced by a significant rise in the serum activities of AST and ALT. ORWP and ORAP pretreatment markedly reduced the serum activities of AST and ALT, especially at the dose of 200 mg/kg. Hence, supplementation with ORWP and ORAP depressed the serum activities of AST and ALT in CCl_4_-intoxicated mice and was effective in undermining the injurious effect of CCl_4_. Bifendate (positive control) at a dose of 200 mg/kg demonstrated the most significant hepatoprotective effect.

**Figure 4:**
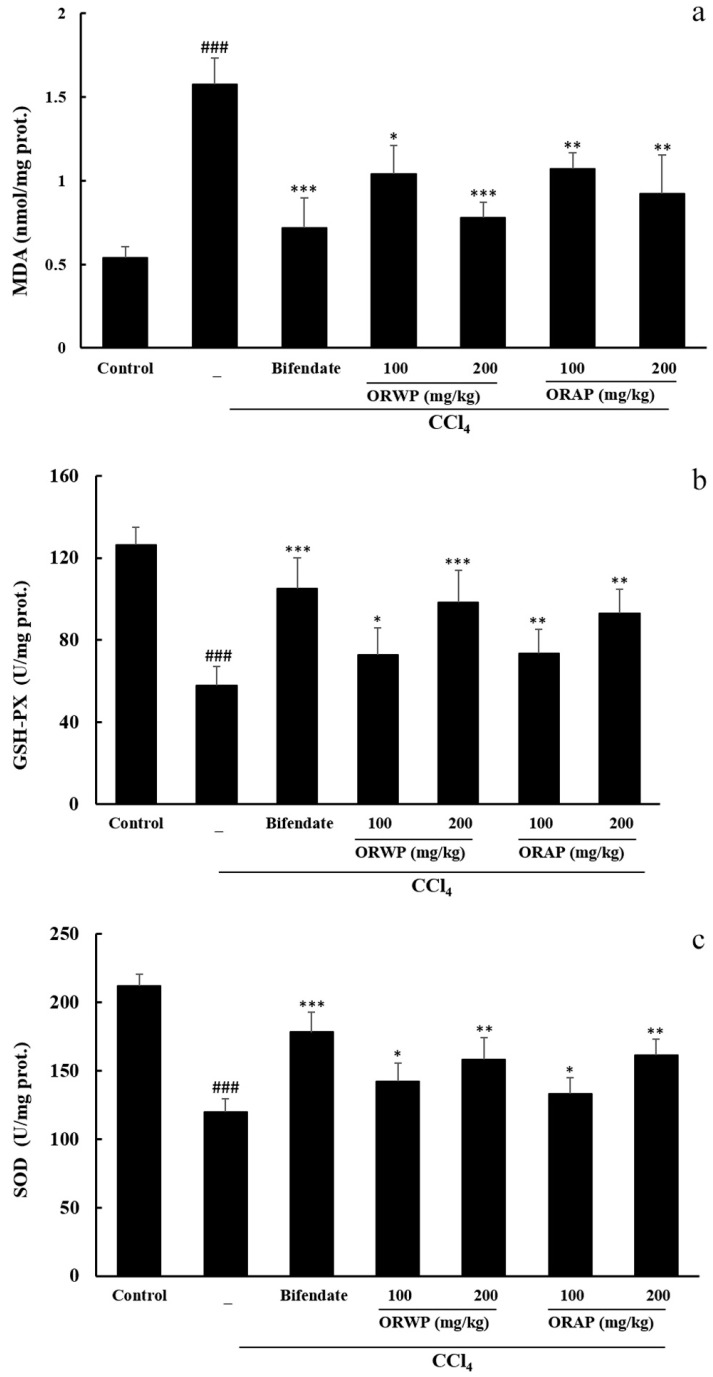
Effects of ORWP and ORAP on hepatic (**a**) MDA content; (**b**) GSH-Px activity and (**c**) SOD activity in CCl_4_-treated mice. The values represent means ± SD of eight mice/group. ^###^ *p* < 0.001 compared with control, *** *p* < 0.001, ** *p* < 0.01 and * *p* < 0.05 versus CCl_4_-intoxicated group.

Hepatic MDA levels indicated lipid peroxidation in the tissue. Prominent elevation of the MDA level in the CCl_4_-intoxicated group was observed when compared with the normal control group. ORWP and ORAP at 100 and 200 mg/kg, dose-dependently, and bifendate suppressed the MDA level (Figure 4a). CCl_4_-induced toxicity caused a significant fall in hepatic activities of GSH-Px and SOD compared to the normal control group which was dose-dependently reversed by pretreatment with ORWP and ORAP at 100 and 200 mg/kg. Treatment with the hepatoprotective drug bifendate also significantly upregulated the hepatic activities of GSH-Px and SOD (Figure 4b,c).

#### 2.3.3. Liver Histopathological Study

**Figure 5:**
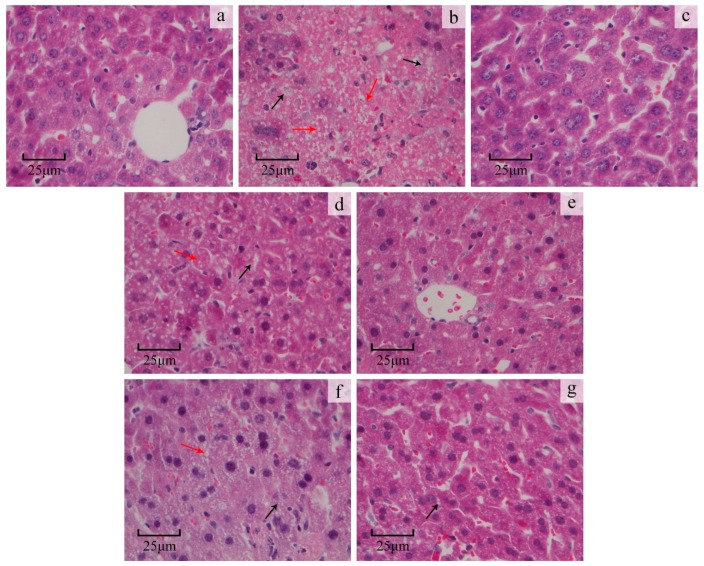
Effects of ORWP and ORAP on liver as disclosed by morphological analysis (×400 H&E staining). (**a**) normal control group; (**b**) CCl_4_-intoxicated group; (**c**) bifendate positive control group (200 mg/kg) + CCl_4_; (**d**) ORWP (100 mg/kg) + CCl_4_; (**e**) ORWP (200 mg/kg) + CCl_4_; (**f**) ORAP (100 mg/kg) + CCl_4_; (**g**) ORAP (200 mg/kg) + CCl_4_. Black arrows indicate necrotic zones; red arrows indicate lipid droplet accumulation.

The normal control group displayed normal liver cells with prominent nuclei and nucleoli, well-preserved cytoplasm and visible central veins (Figure 5a). CCl_4_ treatment induced extensive liver injury characterized by moderate to severe cellular degeneration, hepatocyte necrosis and lipid droplet accumulation (Figure 5b). Bifendate pretreatment effectively protected against CCl_4_-induced liver damage (Figure 5c). ORWP and ORAP pretreatment apparently ameliorated liver damage, as demonstrated by a reduced number of necrotic zones and lipid droplet accumulation (Figure 5d–g). These findings were in accordance with the levels found for serum and hepatic enzymes used as markers.

#### 2.3.4. Study on the Acute Toxicity

ORWP and ORAP exhibited no acute toxicity towards the experimental mice. Additionally, no mortality occurred within 48 h either in the control or treated groups. No gross behavioral differences were found between the groups in the first 24 h.

## 3. Discussion

Reactive oxygen species (ROS) have received considerable attention because of their role in heart diseases, cancer, diabetes and cancer [11,21]. The delicate balance between ROS production and clearance is critical to maintain normal cellular physiology [22]. Antioxidants can delay or reduce substrate oxidation, and protect the body from oxidative damage induced by excessive free radicals [23,24]. However, some commercial synthetic antioxidants like butylated hydroxytoluene, tertiary butylated hydroquinone and gallic acid ester have potential adverse effects [25]. Hence, there is increasing interest in searching for natural antioxidants to replace synthetic antioxidants. Plant, algal and fungal polysaccharide extracts possess free radical scavenging activities [26,27]. In this study, the antioxidant effects of ORWP and ORAP were measured under in vitro and in vivo conditions.

The DPPH free radical has been widely used to evaluate the free radical-scavenging activities of antioxidants [28]. ORWP and ORAP exhibited significant dose-dependent DPPH radical scavenging activities, in keeping with findings on *Ganoderma tsugae* polysaccharides [29].

During the last few decades, lipid peroxidation has attracted considerable research interest [30,31]. In this study, polyunsaturated linoleic acid was oxidized in a water emulsion. The resulting peroxyl (LOO•) and alkoxyl (LOS•) radicals formed preexisting lipid peroxide (LOOH) to initiate lipid peroxidation. Usually, antioxidants inhibit lipid peroxidation by scavenging lipid-derived radicals (LOO• or LOS•) [32]). In our study, ORWP showed stronger activity than ORAP in inhibiting linoleic acid peroxidation. ORWP had an inhibitory activity closer to the strong antioxidant ascorbic acid, at 5 mg/mL, indicating that this polysaccharide may be a good antioxidant.

Hydrogen peroxide formed in tissues through oxidative processes causes toxicity to the cells because it depletes antioxidants, and produces strand breaks in DNA and oxidative degradation of lipids, proteins, carbohydrates and nucleic acids [33]. ORWP and ORAP possessed moderate H_2_O_2_ scavenging activity. At 5 mg/mL, 78.07%, 60.32% and 100% scavenging was brought about by ORWP, ORAP and ascorbic acid, respectively.

It has been reported that the antioxidant effect of a compound may be concomitant with the development of reducing power. The reducing properties were generally associated with the presence of reductones, which have been shown to exert antioxidant action by breaking the free radical chain via donating a hydrogen atom [34]. The polysaccharides from *O. radicata* acted as reductants by effecting the conversion of the Fe^3+^/ferricyanide complex to the Fe^2+^ form, and the Fe^2+^ concentration could be determined by measuring the enhanced formation of Perl’s Prussian blue at 700 nm. Our results suggest that ORWP and ORAP possessed a moderate reducing power, which increased with an increase in the polysaccharide concentration. From the results, it may be considered that ORWP and ORAP are hydrogen donors and might react with free radicals to stabilize and terminate radical chain reactions.

Metal chelating activity is claimed as one of the antioxidant mechanisms, since it reduces the concentration of the transition metal catalyzing lipid peroxidation [35]. According to previous reports, some transition metals can trigger the production of free radicals and magnify the cellular damage [25]. Among the transition metals, ferrum is known as the most important prooxidant due to its high reactivity. The ferrous state of iron can stimulate lipid oxidation by generating reactive free radicals via the Fenton reaction [25]. Hence, it has been recognized that metal ion chelating agents may inhibit lipid oxidation by stabilizing Fe^2+^. Ferrozine can quantitatively form red-colored complexes with Fe^2+^. The metal chelating activity of antioxidants is estimated by their capacity to inhibit the formation of the red-colored ferrozine-Fe^2+^ complex [36]. In the present study, the data on Fe^2+^ chelating activity demonstrated that ORWP and ORAP exhibited moderate chelating activity. Ker et al. [37] showed that the concentration of available hydroxyl groups is instrumental to the chelating ability of polysaccharides from *Agaricus blazei* mycelia. The chelating effect of ORWP and ORAP might be partly due to the Fe^2+^ chelating groups in the structure.

A model has been proposed for the antioxidant ability of carbohydrate polymers [38]. It has been demonstrated that the antioxidant effect of glucans and nonglucan polymers, which are significantly better free radical scavengers than monosaccharides, did not correlate with the type of intrachain linkages, molecular weight or degree of polymer branching, but rather with the monosaccharide composition of the polymer. The weak activity of monosaccharides is attributed to abstraction of the anomeric hydrogen, and the enhanced activity of the polymers to the greater ease of abstraction of anomeric hydrogen from one of the internal monosaccharide units rather than from the reducing end [39]. ORWP and ORAP were mainly composed of mannose, glucose and galactose. The higher antioxidant ability of ORWP than ORAP is likely due to more facile anomeric hydrogen abstraction than ORAP.

CCl_4_ is a well-known hepatotoxic chemical. In the hepatic parenchyma cells, it is metabolized to trichloromethyl radicals (CCl_3_•) by the enzymatic action of cytochrome P450. The trichloromethyl radical reacts rapidly with oxygen to yield trichloromethylperoxy radical (CCl_3_OO•), which is highly reactive and can attack polyunsaturated fatty acids of the cellular membranes, resulting in loss of membrane integrity and leakage of microsomal enzymes [40]. Bifendate (biphenyldicarboxylate) is a synthetic hepatoprotective agent derived from the compound schisandrin C. It is active against a variety of hepatotoxins and has been used as a curative agent for the treatment of hepatitis with minimal observable side effects at the prescribed dosage. Many authors considered bifendate as a hepatoprotective agent against drug-induced liver injuries in animals [3,41]. In our study, bifendate was regarded as a positive control for exploring the hepatoprotective effect of water-soluble and alkali-soluble polysaccharides from *O. radicata* (ORWP and ORAP).

In the present study, we demonstrated for the first time that treatment with ORWP and ORAP could significantly prevent CCl_4_-induced acute liver toxicity in mice. The serum levels of AST and ALT have been used as biochemical markers for acute liver damage [36]. When compared with the normal control group, the mice treated with CCl_4_ alone showed acute liver damage as evidenced by a marked elevation of the serum levels of AST and ALT [34]. It was reported that the leakage of large quantities of enzymes into the blood stream was associated with massive centrilobular necrosis, ballooning degeneration and cellular infiltration of the liver [42]. However, the elevated levels of these enzymes were significantly reduced by pretreatment with ORWP and ORAP, implying that ORWP and ORAP prevent liver damage and suppress the leakage of enzymes through cellular membranes.

The hepatotoxic effects of CCl_4_ are largely due to its active metabolite, which can attack polyunsaturated fatty acids of cell membranes and induce lipid peroxide formation. MDA is an indicator of lipid peroxides. In this study, the increase of MDA in CCl_4_-intoxicated mice indicated excessive free radical formation and enhanced peroxidation leading to hepatic damage. Administration of ORWP and ORAP for 10 consecutive days significantly lowered the MDA level. Free radical scavenging enzymes such as SOD and GSH-Px constitute the primary defense system against ROS. SOD and GSH-Px catalyze respectively, superoxide radical (O_2_^−^) reduction to H_2_O_2_ and O_2_, and H_2_O_2_ reduction to H_2_O and O_2_, thereby preventing hydroxyl radical formation [43]. Results of the present study showed that the activities of hepatic SOD and GSH-Px in CCl_4_-intoxication mice were significantly decreased, indicating elevated oxidative damage to the liver. However, ORWP and ORAP pretreatment raised the levels of hepatic SOD and GSH-Px in CCl_4_-treated mice, suggesting significant hepatoprotective effect which was also confirmed by histopathological studies that revealed a decrease in area of the necrotic zones and lipid droplet accumulation. This might be partly due to free radical scavenging effect, increased antioxidant capability and inhibition of lipid peroxidation. Previous studies suggested that most polysaccharides cannot be digested by humans due to the lack of fiber-degrading enzymes encoded in our genome [11]. Rice et al. [44] showed that soluble β-glucans such as laminarin and scleroglucan can be directly bound and internalized by intestinal epithelial cells and gut associated lymphoid tissue (GALT) cells. We speculate that most of ORWP/ORAP was absorbed in the small intestine, and then effectively protect the mice from CCl_4_-induced hepatic toxicity. The underlying mechanism for the absorption and tissues distribution of ORWP/ORAP in mice has yet to be elucidated.

## 4. Materials and Methods

### 4.1. Materials and Chemicals

Dried fruiting bodies of the mushroom *O. radicata* were purchased from a market in Hebei Province (China). The diagnostic kits for AST (aspartate aminotransferase), ALT (alanine aminotransferase), MDA (malondialdehyde), SOD (superoxide dismutase), and GSH-Px (glutathione peroxidase) were purchased from Nanjing Jiancheng Bioengineering Institute (Nanjing, China). DPPH (1,1-diphenyl-2-picrylhydrazyl), ascorbic acid, linoleic acid, ferrozine (3-(2-pyridyl)-5,6-diphenyl-1,2,4-triazine-4′4′′-disulfonic acid monosodium salt), were purchased from Sigma-Aldrich (Steinheim, Germany). All other chemicals were of analytical grade and were purchased from Beijing Chemical Co. (Beijing, China).

### 4.2. Extraction and Isolation of Polysaccharides

*O. radicata* polysaccharides were isolated following a published procedure [45]. Briefly, dried fruiting bodies of the mushroom (100 g) were crushed into powder using a disintegrator (Baijie, Deqing, China). The powder was extracted twice with 20 vol 95% ethanol (*w/v*) for 2 h to remove lipids. The degreased powders were air-dried, and then extracted with 10 vol of water (*w/v*) at 90 °C for 6 h. The supernatant obtained after centrifugation (9000× *g*, 4 °C, 10 min), and then the residue collected was extracted with 500 mL 0.5 M NaOH solution at room temperature for 5 h. The extract was condensed and precipitated by adding 3 vol 95% ethanol at 4 °C overnight. The precipitate was collected by centrifugation, deproteinated, and then dialysed overnight against distilled water [46]. Finally the deproteinated supernatant was lyophilized to obtain crude *O. radicata* polysaccharides (cORWP/cORAP). The cORWP/cORAP was dissolved in distilled water, and then applied to a 5 cm × 20 cm column of DEAE-cellulose (Sigma, St. Louis, MO, USA), which was eluted at 3.0 mL/min with distilled water. The eluent was concentrated, lyophilized, and then subjected to gel filtration on a Superdex 75 HR 10/30 column (GE Healthcare, Little Chalfont, UK) in 0.15 M NH_4_HCO_3_ buffer (pH 8.5). The eluent was collected automatically and the carbohydrate content was determined. The main fraction was lyophilized to yield ORWP/ORAP which was used for further studies.

### 4.3. Molecular Weight Determination

The molecular weight of ORWP/ORAP was determined by high performance gel permeation chromatography (HPGPC) [45]. The sample solution was applied to an 1100 HPLC system (Agilent, Santa Clara, CA, USA) equipped with a TSK-GEL G3000 PWXL column (7.8 mm × 300 mm, column temperature: 35 °C), and then eluted with 0.05 mol/L NaH_2_PO_4_–Na_2_HPO_4_ solution at a flow rate of 0.8 mL/min. The peaks were detected using a differential refractive index detector. The molecular weight was estimated by reference to the calibration curve constructed with standard dextrans (molecular weights of 738, 5800, 1.22 × 10^4^, 2.37 × 10^4^, 4.8 × 10^4^, 1.0 × 10^5^, 1.86 × 10^5^, 3.8 × 10^5^, and 8.53 × 10^5^).

### 4.4. Preliminary Characterization of ORWP/ORAP

Total carbohydrate content of the polysaccharide was determined by using the authrone-sulfuric acid colorimetric method, with glucose as the standard [47]. The protein content of the polysaccharide was measured by using Bradford’s method [48]. The monosaccharide composition of ORWP/ORAP was analyzed by gas chromatography-mass spectrometer (GC-MS). ORWP/ORAP was firstly hydrolyzed with 2 M H_2_SO_4_ at 100 °C for 6 h. After neutralization with Ba(OH)_2_, the supernatants were collected and lyophilized. The hydrolysates were then converted into their completely acetylated derivatives and analyzed by GC-MS using the method reported by Han et al. [49].

### 4.5. UV and Infrared Spectral Analysis

The UV absorption spectra were obtained with a spectrophotometer (Thermo Scientific, Waltham, MA, USA) in the range of 200–400 cm^−1^ [46]. The infrared spectra of ORWP/ORAP were determined using a fourier transform infrared spectrophotometer (Thermo Nicolet, Waltham, MA, USA) with KBr pellets in a frequency range of 4000–500 cm^−1^.

### 4.6. Analyse of the Antioxidant Activity In Vitro

#### 4.6.1. DPPH Radical Scavenging Activity

Scavenging activity of ORWP/ORAP toward DPPH free radicals was determined using the method of Cheng et al. [43] with slight modifications. Briefly, 150 μL of different concentrations (0–5 mg/mL) of the samples were added to 450 μL of a 0.004% methanol solution of DPPH, and the mixture was shaken thoroughly. After incubation in the dark (30 °C, 30 min), absorbance (A) of the assay mixture was determined at 517 nm. Ascorbic acid was used for comparison:

Percent scavenging of DPPH radical (%) = (A_control_ − A_sample_)/A_control_ × 100 (1)

#### 4.6.2. Inhibitory Effect on Lipid Peroxidation

The inhibitory effect on lipid peroxidation was determined in according to the conjugated diene method with some modifications [29]. Samples at different concentrations (0–5 mg/mL) were prepared, and incubated with 500 μL linoleic acid emulsion (10 mM) in 0.2 M sodium phosphate buffer (pH 6.5) for 15 h at 37 °C. Then, 1.5 mL 60% methanol in deionized water was added, and the absorbance (A) was measured at 234 nm. Ascorbic acid was used for comparison:

Inhibition of lipid peroxidation (%) = (A_control_ − A_sample_)/A_control_ × 100 (2)

#### 4.6.3. Hydrogen Peroxide Scavenging Activity

The ability of ORWP/ORAP to scavenge hydrogen peroxide was measured as reported by Ganie et al. [21], with minor modification. A solution of H_2_O_2_ (0.3%) was prepared in phosphate buffer (0.1 M, pH 7.4). ORWP/ORAP was dissolved in distilled water at 1.0, 2.0, 3.0, 4.0, and 5.0 mg/mL. The samples were mixed with 40 μL H_2_O_2_ solution, and the mixtures were shaken vigorously and incubated at room temperature for 10 min. The absorbance (A) was determined at 230 nm. Ascorbic acid was used for comparison:

Scavenging of H_2_O_2_ (%) = (A_control_ − A_sample_)/A_control_ × 100 (3)

#### 4.6.4. Reducing Power

Reducing power was evaluated with a slight modification of the published method [50]. One milliliter of sample, 1.0 mL phosphate buffer (0.2 M, pH 6.6) and 1.0 mL 0.1% potassium ferricyanide were incubated in a water bath (50 °C, 20 min). After 1 mL of 10% trichloroacetic acid was added, the mixture was centrifuged (3000 rpm, 10 min). The supernatant (1 mL) was mixed with 1 mL distilled water and 0.2 mL 0.3% ferric chloride, and the absorbance was measured at 700 nm. All assays were carried out in triplicate. Ascorbic acid was used for comparison.

#### 4.6.5. Ferrous Ion Chelating Activity

The ferrous ion chelating activity was investigated according to the reported method [51]. Briefly, the reaction mixture, containing 200 μL sample (0–5 mg/mL), 10 μL ferrous chloride (FeCl_2_), 40 μL ferrozine solution (5 mM) and 550 μL distilled water, was shaken well and incubated for 10 min at room temperature. The absorbance (A) was determined at 562 nm. The chelating agent ethylenediamine tetraacetic acid disodium salt (EDTA) was used for comparison:

Fe^2+^ ion chelating activity (%) = (A_control_ − A_sample_)/A_control_ × 100 (4)

### 4.7. Animal Experiments

#### 4.7.1. Animals

Male Kunming mice (weighing 18–22 g) were provided by Xinglong Experimental Animal Breeding Factory (Beijing, China). The animals were allowed to acclimatize for 7 days before the experiment. They had access to a standard diet and water *ad libitum* and were maintained at 25 ± 2 °C, and 55% ± 5% relative humidity with a 12 h light/dark cycle. All animal studies were in strict accordance with the Chinese legislation on the use and care of laboratory animals.

#### 4.7.2. In Vivo Hepatoprotective Activity

The mice were randomly allocated into seven groups of eight animals each. In the normal control group and CCl_4_-intoxication group, animals were given a single dose of distilled water (0.2 mL) by intragastric (i.g.) gavage once daily for 10 days. The positive group was treated with bifendate (200 mg/kg in 0.5% sodium carboxymethyl cellulose, i.g.) once daily for 10 days. In the four experimental groups, the mice were pretreated with ORWP and ORAP (100 and 200 mg/kg, i.g.), once daily for 10 consecutive days. On the 11th day, all mice except those in the normal control group were given a CCl_4_/peanut oil mixture (1%, 5 mL/kg, i.g.) 2 h after the last administration of bifendate, ORWP and ORAP, while the normal control group received peanut oil alone. Sixteen hours later, the animals were weighed and sacrificed under light ethyl ether anesthesia. The liver was weighed and homogenized (10% *w/v*) in ice-cold physiological saline. The supernatant obtained after centrifugation (8000 rpm, 10 min) was stored at −20 °C. The hepatosomatic index (HI) was calculated as liver weight/body weight × 1000%.

#### 4.7.3. Biochemical Assays

After blood collection, serum was obtained by centrifugation (5000 rpm, 10 min). The activities of ALT and AST in the serum were measured using commercial kits, and the results were expressed as units per liter (IU/L). The activity of hepatic superoxide dismutase (SOD), glutathione peroxidase (GSH-Px) and malondialdehyde (MDA), considered as indexes of antioxidant status of liver tissues, was assayed using commercial kits, and normalized with reference to protein. The protein concentration was measured by using Bradford’s method [48].

#### 4.7.4. Histopathological Study on the Liver

A small piece of the liver was preserved in 10% neutral buffered formaldehyde solution, processed and embedded in paraffin. Sections (4 μm thickness) were cut, stained with hematoxylin-eosin (H&E), and evaluated for pathological changes under the light microscope.

#### 4.7.5. Study on the Acute Toxicity

Male Kunming mice were randomly divided into five groups of 8 animals each. In the control group, mice were given distilled water by gavage. In the experimental groups, mice were given ORWP and ORAP (500 and 1000 mg/kg respectively). The animals were observed continuously in the first 24 h for any gross behavioral changes and toxic symptoms, and in the first 48 h for mortality.

### 4.8. Statistical Analysis

All tests were performed in triplicate and the results were expressed as mean ± SD. Statistical analysis was performed using IBM SPSS Statistical software (version 20, IBM, Armonk, NY, USA) to determine the difference between the groups. A *p* value < 0.05 was considered to be statistically significant.

## 5. Conclusions

In the present study, the in vitro antioxidant activity and in vivo hepatoprotective activity of ORWP and ORAP were investigated. As demonstrated in our previous studies, ORWP and ORAP manifested potent antioxidant activity in various in vitro assays. Additionally, the hepatoprotective effect showed good correlation with antioxidant activity. The current study also demonstrated that the administration of ORWP and ORAP significantly decreased serum ALT and AST levels, inhibited MDA formation and enhanced antioxidant enzyme activities. The results suggested that the mushroom *O. radicata* may be an excellent source of antioxidant and hepatoprotective polysaccharides, which could be developed as a natural functional food ingredient or a novel nutraceutical to enhance health.

## Figures and Tables

**Table 1:** General compositions, constituent monosaccharides and molecular weights of ORWP and ORAP.

Property/Component	ORWP	ORAP
Molecular weight (Da)	1.73 × 10^5^	1.15 × 10^4^
Carbohydrate (wt %)	95.3	97.4
Protein (wt %)	ND	ND
Components of monosaccharide (mol %)		
Ribose	0.15	0.16
Rhamnose	0.32	0.51
Arabinose	1.02	1.02
Xylose	2.45	3.31
Mannose	15.74	18.41
Glucose	65.85	72.35
Galactose	14.47	4.24

**Table 2:** Effects of ORWP and ORAP on body weight, liver weight and hepatosomatic index (HI) in CCl_4_-treated mice.

Treatment	Dose (ORWP/ORAP)	Body wt. (g)	Liver wt. (g)	HI (%)
Normal		29.13 ± 3.22	1.23 ± 0.20	42.24 ± 2.87
CCl_4_ alone		29.07 ± 1.50	1.54 ± 0.09 ^##^	53.25 ± 3.48 ^###^
CCl_4_ + bifendate	200 mg/kg	29.86 ± 1.10	1.39 ± 0.12 **	46.66 ± 4.12 ***
CCl_4_ + ORWP	100 mg/kg	29.29 ± 2.71	1.46 ± 0.16	49.87 ± 3.36 *
CCl_4_ + ORWP	200 mg/kg	29.41 ± 1.61	1.43 ± 0.08 *	48.50 ± 1.92 **
CCl_4_ + ORAP	100 mg/kg	29.23 ± 1.21	1.42 ± 0.16 *	48.44 ± 4.53 **
CCl_4_ + ORAP	200 mg/kg	29.18 ± 2.22	1.39 ± 0.17 **	47.46 ± 2.98 ***

Mice were randomized into seven groups: control group (normal control), CCl_4_-intoxicated (CCl_4_ only group), bifendate pretreatment at 200 mg/kg plus CCl_4_-intoxication (CCl_4_ + bifendate group), ORWP pretreatment at 100 mg/kg plus CCl_4_-intoxication (CCl_4_ + ORWP group), ORWP pretreatment at 200 mg/kg plus CCl_4_-intoxication (CCl_4_ + ORWP group), pretreatment with ORAP at 100 mg/kg plus CCl_4_-intoxication (CCl_4_ + ORAP group), ORAP pretreatment at 200 mg/kg plus CCl4-intoxication (CCl_4_ + ORAP group). CCl_4_-induced hepatotoxic mice were given a CCl_4_/peanut oil mixture by intragastric (i.g.) gavage (1%, 5 mL/kg), while the normal control group received peanut oil alone. The values represent means ± SD of eight mice/group. ^###^ *p* < 0.001, ^##^ *p* < 0.01 compared with normal control; *** *p* < 0.001, ** *p* < 0.01 and * *p* < 0.05 compared with the CCl_4_-intoxication group.
